# Supplementary material for: Faunal communities mediate the effects of plant richness, drought, and invasion on ecosystem multifunctional stability
Source: Commun Biol. 2022 Jun 1;5:527. doi: 10.1038/s42003-022-03471-0 (PMC9159989; doi:10.1038/s42003-022-03471-0)
Supplement: Supplementary file 2 — Reporting Summary [file 42003_2022_3471_MOESM2_ESM.pdf]

nature portfolio

Corresponding author(s): Yuan Ge

Last updated by author(s): Apr 12, 2022

Reporting Summary

Nature Portfolio wishes to improve the reproducibility of the work that we publish. This form provides structure for consistency and transparency in reporting. For further information on Nature Portfolio policies, see our Editorial Policies and the Editorial Policy Checklist.

Statistics

For all statistical analyses, confirm that the following items are present in the figure legend, table legend, main text, or Methods section.

n/a	Confirmed	
☐	☒	The exact sample size (*n*) for each experimental group/condition, given as a discrete number and unit of measurement
☒	☐	A statement on whether measurements were taken from distinct samples or whether the same sample was measured repeatedly
☐	☒	The statistical test(s) used AND whether they are one- or two-sided<br>*Only common tests should be described solely by name; describe more complex techniques in the Methods section.*
☒	☐	A description of all covariates tested
☒	☐	A description of any assumptions or corrections, such as tests of normality and adjustment for multiple comparisons
☐	☒	A full description of the statistical parameters including central tendency (e.g. means) or other basic estimates (e.g. regression coefficient) AND variation (e.g. standard deviation) or associated estimates of uncertainty (e.g. confidence intervals)
☐	☒	For null hypothesis testing, the test statistic (e.g. *F*, *t*, *r*) with confidence intervals, effect sizes, degrees of freedom and *P* value noted<br>*Give P values as exact values whenever suitable.*
☒	☐	For Bayesian analysis, information on the choice of priors and Markov chain Monte Carlo settings
☒	☐	For hierarchical and complex designs, identification of the appropriate level for tests and full reporting of outcomes
☒	☐	Estimates of effect sizes (e.g. Cohen's *d*, Pearson's *r*), indicating how they were calculated

Our web collection on statistics for biologists contains articles on many of the points above.

Software and code

Policy information about availability of computer code

Data collection	Protein representative faunal mitochondrial or plant plastid coding genes were obtained by searching plant or faunal taxonomies from NCBI protein database (https://www.ncbi.nlm.nih.gov/protein/) with Edirect software (https://www.ncbi.nlm.nih.gov/books/NBK179288/). The Linux codes for processing the protein sequences were submitted to GitHub (https://github.com/YuanGe-Lab/JZW_2022/tree/main/linux). All other analyses were conducted using R-4.0.3 (https://www.r-project.org). The R codes and examples solving the permutation test for the significance of effects derived from SEMs that based on multidimensional similarity (or distance) were submitted to GitHub (https://github.com/YuanGe-Lab/JZW_2022/tree/main/R).
Data analysis	Protein representative faunal mitochondrial or plant plastid coding genes were obtained by searching plant or faunal taxonomies from NCBI protein database (https://www.ncbi.nlm.nih.gov/protein/) with Edirect software (https://www.ncbi.nlm.nih.gov/books/NBK179288/). The Linux codes for processing the protein sequences were submitted to GitHub (https://github.com/YuanGe-Lab/JZW_2022/tree/main/linux). All other analyses were conducted using R-4.0.3 (https://www.r-project.org). The R codes and examples solving the permutation test for the significance of effects derived from SEMs that based on multidimensional similarity (or distance) were submitted to GitHub (https://github.com/YuanGe-Lab/JZW_2022/tree/main/R). The executable codes that support our findings, figures and tables are available at GitHub (https://github.com/YuanGe-Lab/JZW_2022)

For manuscripts utilizing custom algorithms or software that are central to the research but not yet described in published literature, software must be made available to editors and reviewers. We strongly encourage code deposition in a community repository (e.g. GitHub). See the Nature Portfolio guidelines for submitting code & software for further information.

Data

Policy information about availability of data

All manuscripts must include a data availability statement. This statement should provide the following information, where applicable:

- Accession codes, unique identifiers, or web links for publicly available datasets
- A description of any restrictions on data availability
- For clinical datasets or third party data, please ensure that the statement adheres to our policy

The datasets generated and/or analysed during the current study are available at GitHub (https://github.com/YuanGe-Lab/JZW_2022).

Field-specific reporting

Please select the one below that is the best fit for your research. If you are not sure, read the appropriate sections before making your selection.

☒ Life sciences ☐ Behavioural & social sciences ☐ Ecological, evolutionary & environmental sciences

For a reference copy of the document with all sections, see nature.com/documents/nr-reporting-summary-flat.pdf

Life sciences study design

All studies must disclose on these points even when the disclosure is negative.

Sample size	To test whether, and how, plant richness, drought, and plant invasion will affect ecosystem multifunctional stability by modulating community compositional stability, a 3-year experiment was performed by exposing 270 plant communities, with four levels of plant richness (1, 2, 4, and 8 species; with 16, 10, 10, and 9 replicates, respectively), to drought (non, moderate, and intensive drought; treated numerically as 0, 1 and 2 during data analyses) and exotic plant invasion (non invasion and invasion; 0 and 1).
Data exclusions	no data were excluded from the analysis.
Replication	To test whether, and how, plant richness, drought, and plant invasion will affect ecosystem multifunctional stability by modulating community compositional stability, a 3-year experiment was performed by exposing 270 plant communities, with four levels of plant richness (i.e., 16 monocultures, 10 two-species mixtures, 10 four-species mixtures, 9 eight-species mixtures with 6 replicates in each), to drought (non, moderate, and intensive drought; treated numerically as 0, 1 and 2 during data analyses) and exotic plant invasion (non invasion and invasion; 0 and 1). We calculated the compositional stability of plants, litter-faunal, and soil-faunal communities based on both their species abundances and phylogenetic traits, and also evaluated the stability of ecosystem multifunctionality, based on 14 function-related variables that relate to biomass production (aboveground and belowground plant biomass, light interception efficiency, and litter- and soil-fauna abundances), soil properties (soil carbon, nitrogen, phosphorus, and glomalin related soil protein (GRSP)), or processes (litter decomposition rate, and activities of β-glucosidase, protease, nitrate reductase, and dehydrogenase). Then, we assessed three aspects of stability, i.e., invariability against environmental stochasticity, drought resistance, and invasion resistance, for both community stability and multifunctional stability. Finally, the direct and indirect effects of plant richness, drought, and invasion on ecosystem multifunctional stability (all aspects), were assessed using structural equation modelings (SEMs). The overall setup and analyses allowed us to test the following hypotheses: 1) Plant richness increases ecosystem multifunctional stability ; 2) Drought/invasion decreases multifunctional stability (invariability, and resistance to invasion/drought); 3) Plant, litter-, and soil-faunal community stability mediate the effects of plant richness, drought, and invasion on multifunctional stability.
Randomization	Monocultures of each species (16 total), and random mixtures of 2, 4 or 8 species (with 16, 10, 10, or 9 distinct assemblages, respectively) were designed, creating a complete set of 45 different herbaceous plant assemblages (pots) in total. Each plant assemblage was replicated 6 times, for a total of 270 pots. To eliminate the non-random effects during the one-year development of the 270 pots, their distributions were randomized, such that not all replicates of an assemblage were next to each other. After one-year of growth, 6 random complete sets (45 pots) were randomly exposed to three levels of drought intensities. Nine months after the introduction of drought stress, 2 complete sets (45 pots) of each drought treatment we randomly exposed (invasion) or not exposed (non invasion) to the invasive species Symphyotrichum subulatum. Such randomizations during this study were addressed in the method section and illustrated in Figure 1.
Blinding	The collected samples (plant, soil and litter) were labeled with meaningless letters to blind investigators from detailed sample information.

Reporting for specific materials, systems and methods

We require information from authors about some types of materials, experimental systems and methods used in many studies. Here, indicate whether each material, system or method listed is relevant to your study. If you are not sure if a list item applies to your research, read the appropriate section before selecting a response.

Materials & experimental systems

n/a	Involved in the study	
☒	☐	Antibodies
☒	☐	Eukaryotic cell lines
☒	☐	Palaeontology and archaeology
☒	☐	Animals and other organisms
☒	☐	Human research participants
☒	☐	Clinical data
☒	☐	Dual use research of concern

Methods

n/a	Involved in the study	
☒	☐	ChIP-seq
☒	☐	Flow cytometry
☒	☐	MRI-based neuroimaging
