# Supplementary material for: Faunal communities mediate the effects of plant richness, drought, and invasion on ecosystem multifunctional stability
Source: Commun Biol. 2022 Jun 1;5:527. doi: 10.1038/s42003-022-03471-0 (PMC9159989; doi:10.1038/s42003-022-03471-0)
Supplement: Supplementary file 3 — Supplementary Information [file 42003_2022_3471_MOESM3_ESM.pdf]

Supplementary Materials for

Faunal communities mediate the effects of plant richness, drought, and invasion on ecosystem multifunctional stability

Authors

Zhongwang Jing[1,2]†, Jiang Wang[3]† , Yi Bai[3], Yuan Ge[1,2]*

Affiliations

[1]State Key Laboratory of Urban and Regional Ecology, Research Center for Eco-Environmental Sciences, Chinese Academy of Sciences, Beijing 100085, China.

[2]University of Chinese Academy of Sciences, 100049 Beijing, China.

[3]School of Life Sciences, Taizhou University, Taizhou 318000, China.

***To whom correspondence may be addressed. Email: yuange@rcees.ac.cn**

†These authors contributed equally to this work.

This file contains **Supplementary method**, **Supplementary TableS 1-4** and **Supplementary Figures S1-5**.

Supplementary method

Estimation of the relative contribution of plant species (or faunal Orders) to the relationship between community stability and ecosystem multifunctional stability.

The contribution of plant species (or their random combinations) to the relationship between plant community stability and ecosystem multifunctional stability (invariability, drought resistance, or invasion resistance), was estimated by conducting following procedures:

1) The original relationship between the stability (invariability, drought resistance and invasion resistance) of original plant community (with no plant species removed) and ecosystem multifunctionality was calculated as the correlation (Pearson's R) between their pairwise similarities (**Supplementary Figure 1**).
2) We randomly removed 1 or a group of (random combinations of 2, 3 or 4 species from 16 plant species) plant species from the original community to imitate the null plant community. Then, the null relationship between the stability (invariability, drought resistance and invasion resistance) of null plant community and ecosystem multifunctionality was calculated.
3) Change (decrease) of the relationship between the community stability and ecosystem multifunctional satiability after the random remove of plant species (or their random combinations) was calculated as the difference between the original relationship and the null relationship.
4) The relative contributions of plant species (or their random combinations) were calculated as the percentage decrease of community-multifunctional stability relationship after the remove of plant species, compared to the original relationship.

Such estimation procedures were also applied to litter/soil faunal communities, but that the species remove was based on faunal Order level.

Table S1 Direct effects of manipulated factors and community stability on the stability of individual functions, derived from structural equation models (SEMs; with identical structures of those in Figure 3) based on three stability aspects (a, invariability; b, drought resistance; c, invasion resistance)

	Direct effects of					
	Plant richness	Drought	Invasion	Plant community stability	Litter-faunal community stability	Soil-faunal community stability
**a**	**on invariability of individual functions**					
Aboveground biomass	**-0.11 ***	0.04	**-0.14 ***	**0.85 *****	0.03	0.02
Light interception efficiency	**0.22 *****	**-0.2 ****	0.04	**0.21 *****	-0.01	-0.01
Belowground biomass	**0.21 ****	-0.08	**0.2 ****	**0.26 *****	-0.02	-0.05
Soil-fauna abundance	0.01	0.02	0.02	0.05	0.04 ^	**0.64 *****
Litter-fauna abundance	0.07	**-0.11 ***	-0.05	-0.01	**0.57 *****	0.01
Soil carbon	**0.15 ****	**-0.24 *****	**-0.09 ***	0.04	0.04	0
Soil phosphorus	**0.11 ^**	**-0.24 *****	0.03	0	**0.08 ***	-0.01
Soil nitrogen	**-0.17 ****	0.08	**-0.44 *****	0.05	-0.03	0
GRSP	**0.2 ****	0.08	-0.07	**0.08 ***	-0.01	0.03
Litter decomposition rate	0.03	-0.04	0.09	**0.22 *****	0.06 ^	**0.11 *****
β-glucosidase	0.08	-0.03	-0.06	0.01	0.03	**0.1 ****
Protease	-0.06	**-0.15 ***	**-0.1 ^**	**0.07 ***	0	**0.16 *****
Nitrate reductase	**0.12 ***	-0.09	0.06	0.01	0.03	**0.1 ****
Dehydrogenase	**0.21 *****	0.1 ^	-0.01	-0.04	**0.09 ****	0.04
**b**	**on drought resistance of individual functions**					
Aboveground biomass	-0.01		**-0.15 ****	**0.8 *****	0.02	0.01
Light interception efficiency	**0.2 ****		0.04	**0.24 *****	0.01	0.04
Belowground biomass	**0.2 ****		**0.21 ****	**0.25 *****	0	0.02
Soil-fauna abundance	0.03		0.05	0.05	0.02	**0.59 *****
Litter-fauna abundance	**0.12 ***		-0.04	-0.06 ^	**0.57 *****	0.01
Soil carbon	-0.08 ^		0.04	-0.01	**0.07 ***	0.01
Soil phosphorus	-0.05		-0.08 ^	0.05	**0.08 ***	0.05 ^
Soil nitrogen	**-0.11 ***		**-0.51 *****	-0.02	**-0.05 ^**	0.02
GRSP	0.03		**-0.12 ***	0	0	0.06 ^
Litter decomposition rate	**0.24 ****		0.03	0.04	**0.11 ****	**0.13 *****
β-glucosidase	0.06		-0.02	0.03	-0.01	0.05 ^
Protease	0.04		-0.09 ^	0.01	-0.03	**0.11 *****
Nitrate reductase	0.07		0.05	0	0	**0.07 ***
Dehydrogenase	**0.19 ****		-0.01	0.01	**0.06 ***	**0.07 ***
**c**	**on invasion resistance of individual functions**					
Aboveground biomass	0.02	0.08		**0.77 *****	0.03	-0.01
Light interception efficiency	**0.2 ***	**-0.2 *****		**0.24 *****	**-0.08 ****	-0.03
Belowground biomass	**0.18 ****	-0.05		**0.3 *****	-0.03	-0.05 ^
Soil-fauna abundance	0.01	0.08		0.05	0	**0.61 *****

Litter-fauna abundance	-0.01	**-0.17** *	0.01	**0.51** ***	0.02
Soil carbon	**0.14** **	**-0.22** **	0	-0.01	0.05
Soil phosphorus	0.07	**-0.21** **	0.06 ^	**0.06** *	-0.02
Soil nitrogen	**0.15** *	**-0.54** ***	**-0.11** **	-0.05 ^	-0.01
GRSP	**0.16** **	0.11 ^	**0.1** **	-0.04	0.06 ^
Litter decomposition rate	0.04	-0.05	**0.26** ***	0.02	**0.08** *
β-glucosidase	0.07	-0.06	0.05 ^	0.03	0
Protease	0.03	**-0.23** ***	0.01	0.01	0.04
Nitrate reductase	**0.12** *	-0.05	0.02	-0.01	**0.09** **
Dehydrogenase	**0.12** *	0.11 ^	-0.03	**0.1** **	0.06 ^

Notes: Symbols after effects indicate significance levels (*** $p < 0.001$, ** $p < 0.01$, * $p < 0.05$, ^ $p < 0.1$, no symbol $p > 0.1$. Mantel-like permutation test with 1,000 randomizations). GRSP, glomalin related soil protein. Blue, positive effect with p less than 0.1; Red, negative effect with p less than 0.1.

Table S2 Deletion of paths along the cascading routes, from manipulated factors (plant richness, drought and invasion)–via the stability of plant, litter-, and soil-faunal communities–to ecosystem multifunctional stability, destabilized the structural equation modelings (SEMs in Figure 2) based on different stability aspects (invariability, SEM 1; drought resistance, SEM 2; invasion resistance, SEM 3)

SEMs		No paths deleted	**Goodness of fit after deleting a single path in SEMs**											
			PR to PL	PR to LF	PR to SF	I to PL	I to LF	I to SF	D To PL	D To LF	D to SF	PL to EMFS	LF to EMFS	SF to EMFS
SEM 1 (**Figure 2a**)	$\chi2$	**0.056**	715.4	**7.3**	24.4	10.9	12.0	57.2	**1.2**	37.4	115.2	140.5	58.8	76.0
	df	**4**	5	**5**	5	5	5	5	**5**	5	5	5	5	5
	p	**1.000**	***	**0.20**	***	0.05	*	***	**0.94**	***	***	***	***	***
SEM 2 (**Figure 2b**)	$\chi2$	**4.416**	744.3	10.4	28.3	15.7	41.9	66.4				172.8	116.6	216.5
	df	**2**	3	3	3	3	3	3				3	3	3
	p	**0.110**	***	*	***	**	***	***				***	***	***
SEM 3 (**Figure 2c**)	$\chi2$	**2.332**	598.5	10.0	30.7				**2.8**	20.3	171.6	149.5	17.1	67.3
	df	**2**	3	3	3				**3**	3	3	3	3	3
	p	**0.312**	***	*	***				**0.79**	***	***	***	***	***

Notes: *** $p < 0.001$, ** $p < 0.01$, and * $p < 0.05$ indicate deletion of a path will generate significantly unstable modelings. PR, plant richness; D, drought; I, invasion; PL, plant community stability; LF, litter- faunal community stability; SF, soil-faunal community stability; EMFS, ecosystem multifunctional stability.

Table S3 Native species pool used in this study

No.	Plant species	Order	Family	Dominant species	Life form	Description	Height (cm)	Leaf habit
1	*Persicaria filiformis*	*Caryophyllales*	*Polygonaceae*	no	Perennial	Erect herb	50-80	(long) oval
2	*Achyranthes aspera*	*Caryophyllales*	*Amaranthaceae*	yes	Perennial	Herb	70-120	(lanceolate) elliptic
3	*Solanum nigrum*	*Solanales*	*Solanaceae*	yes	Annual	Erect herb	25-100	ovate
4	*Penthorum chinense*	*Saxifragales*	*Penthoraceae*	no	Perennial	Herb	40-65	narrow lanceolate
5	*Sesbania cannabina*	*Fabales*	*Fabaceae*	yes	Annual	Herb (legumes)	300-350	linear oblong
6	*Patrinia scabiosifolia*	*Dipsacales*	*Caprifoliaceae*	yes	Perennial	Erect herb	30-100	(ovate/elliptic) lanceolate
7	*Eclipta prostrata*	*Asterales*	*Asteraceae*	no	Annual	Herb	60	(oblong) lanceolate
8	*Persicaria chinensis*	*Caryophyllales*	*Polygonaceae*	no	Perennial	Erect herb	70-100	(long) ovate
9	*Bidens pilosa*	*Asterales*	*Asteraceae*	no	Annual	Erect herb	30-100	(ovate) oval
10	*Perilla frutescens*	*Lamiales*	*Lamiaceae*	no	Annual	Erect herb	30-200	broad ovate
11	*Artemisia stolonifera*	*Asterales*	*Asteraceae*	yes	Perennial	Herb	50-120	oval obovate
12	*Justicia procumbens*	*Lamiales*	*Acanthaceae*	no	Annual	Herb	50	elliptic oblong
13	*Polygonum lapathifolium*	*Caryophyllales*	*Polygonaceae*	no	Annual	Erect herb	40-90	(broad) lanceolate
14	*Lolium perenne*	*Poales*	*Poaceae*	no	Perennial	Ryegrass	80-100	blade linear
15	*Cichorium intybus*	*Asterales*	*Asteraceae*	yes	Perennial	Erect herb	40-100	long elliptic oblanceolate
16	*Medicago sativa*	*Fabales*	*Fabaceae*	no	Perennial	Erect herb (legumes)	30-100	ovate lanceolate

Table S4 Nutritional element contents of vermiculite-compost, soil, and soil-compost mixture

	Vermiculite-compost									
	N	P	K	Mg	Fe	Mn	Zn	Cu	B	Mo
g/kg	6.842	1.318	1.720	0.055	0.044	0.013	0.004	0.007	0.002	0.008

	Soil		Soil-compost mixture	
	Total N	Total P	Total N	Total P
g/kg	0.76 ± 0.10	0.22 ± 0.05	4.61 ± 0.46	0.80 ± 0.19

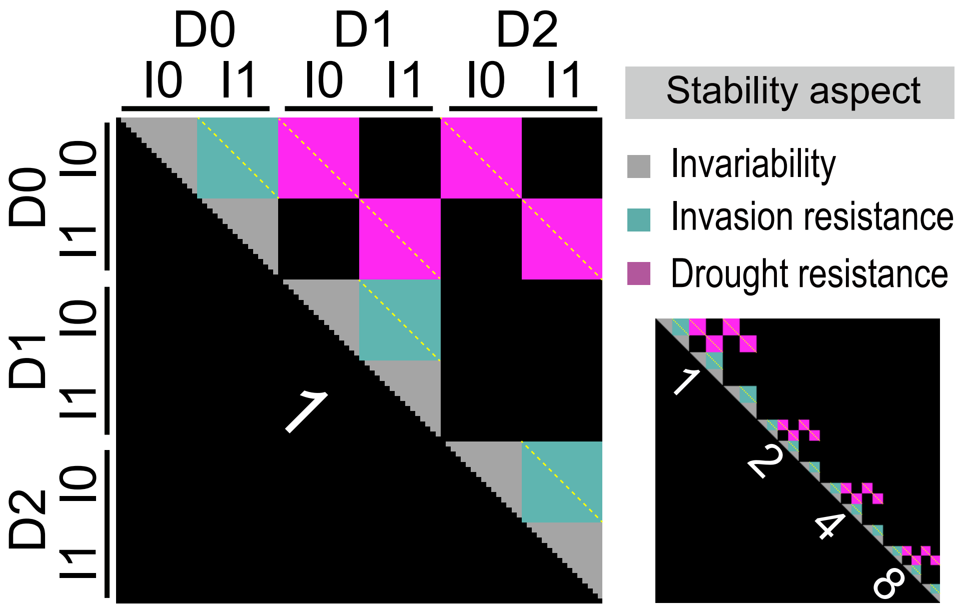

Supplementary Figure 1 An example (plant richness = 1) showing the subsets of each symmetric similarity matrix used for assessing three different aspects of stability (invariability, drought resistance and invasion resistance). I0, non-invasion; I1, invasion; D0, non-drought; D1, moderate-drought; D2, intensive-drought. The subset data for each of other levels of plant richness (2, 4, or 8 species) were extracted similarly. Data marked by the dashed line were those in strict one-to-one correspondence manner and used for generating **Supplementary Figure 2**.

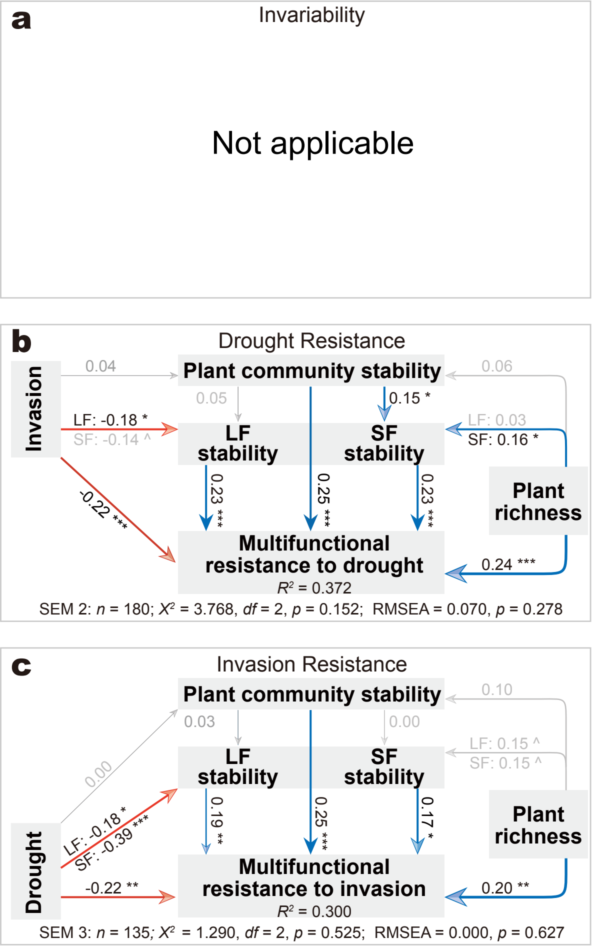

Supplementary Figure 2 Structural equation modelings (SEMs; in strict one-to-one correspondence manner) showing the direct and indirect effects of plant richness, drought, and invasion on ecosystem multifunctional stability**. a**) The modeling for multifunctional invariability (against stochastic fluctuations) was not applicable with the dataset in this study, using the strict one-to-one correspondence method. **b**) Direct and indirect effects on drought resistance calculated as the similarity between drought (moderate and intensive-drought) and non-drought. **c**) Direct and indirect effects on invasion resistance calculated as the similarity between invasion and non-invasion. LF, litter-faunal community; SF, soil-faunal community. n, number of observations (**Supplementary Figure 1**); χ^2, Chi-square; *df*, degree of freedom; *p*, probability level; RMSEA, root-mean squared error of approximation are the goodness-of-fit statistics for each model. Significance of effects derived from SEMs are indicated by symbols (* $p < 0.05$, ** $p < 0.01$, *** $p < 0.001$, ^ $p < 0.1$, and no symbol $p > 0.1$). Blue, positive effect; Red, negative effect.

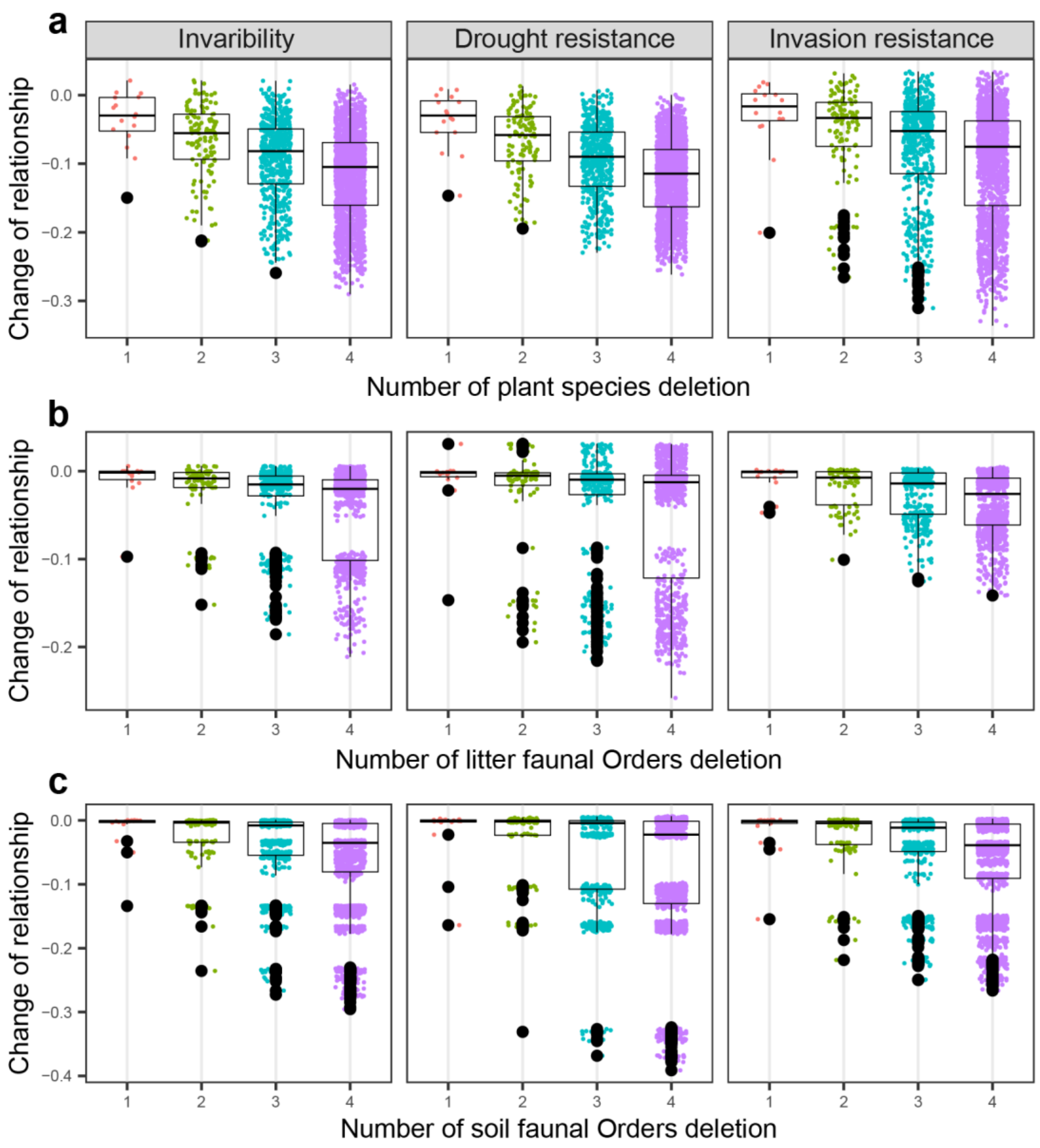

Supplementary Figure 3 Change of relationship between community stability and ecosystem multifunctional stability after random remove of single (1) or a group of (2, 3 and 4) plant species (**a**), litter faunal Orders (**b**) or soil faunal Orders (**c**). X-axis, number of plant species (or faunal Orders) removed from the originally composed plant (or faunal) communities. Y-axis, change of the relationship between the community stability (invariability, drought resistance and invasion resistance) and ecosystem multifunctional satiability after random remove of plant species or faunal Orders, calculated as the difference between the original correlation (between the stability of the original plant/faunal community and the stability of multifunctionality) and the null correlation (between the stability of the plant/fauna-removed community and the stability of multifunctionality). Box-plot: lines indicated 25%, 50% and 75% percentiles; outliers (> 1.5x interquartile range) were indicated by black points.

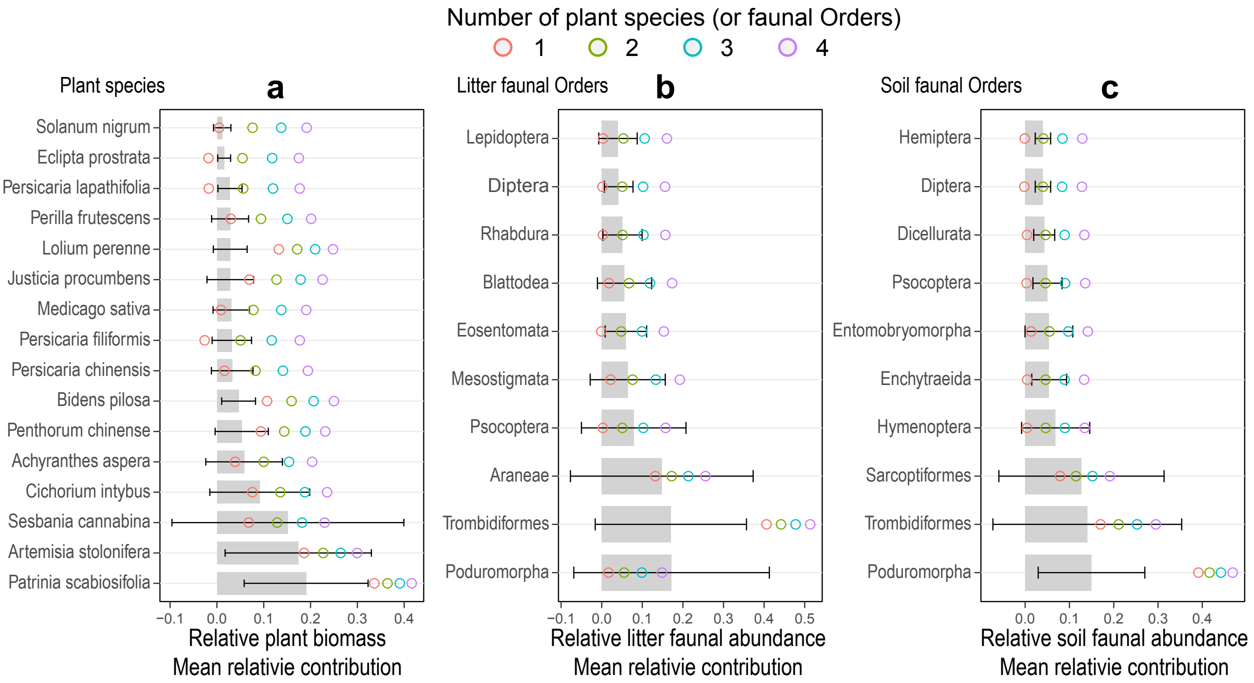

Supplementary Figure 4 Relative contribution of plant species (**a**), litter faunal Orders (**b**), and soil faunal Orders (**c**) to the relationship between community stability and ecosystem multifunctional stability. Circle point indicated the relative contribution of plant species (or faunal Orders) to the relationship between community stability and ecosystem multifunctional stability. Bars and error bars were the means and standard deviations of plant biomass or faunal abundances.

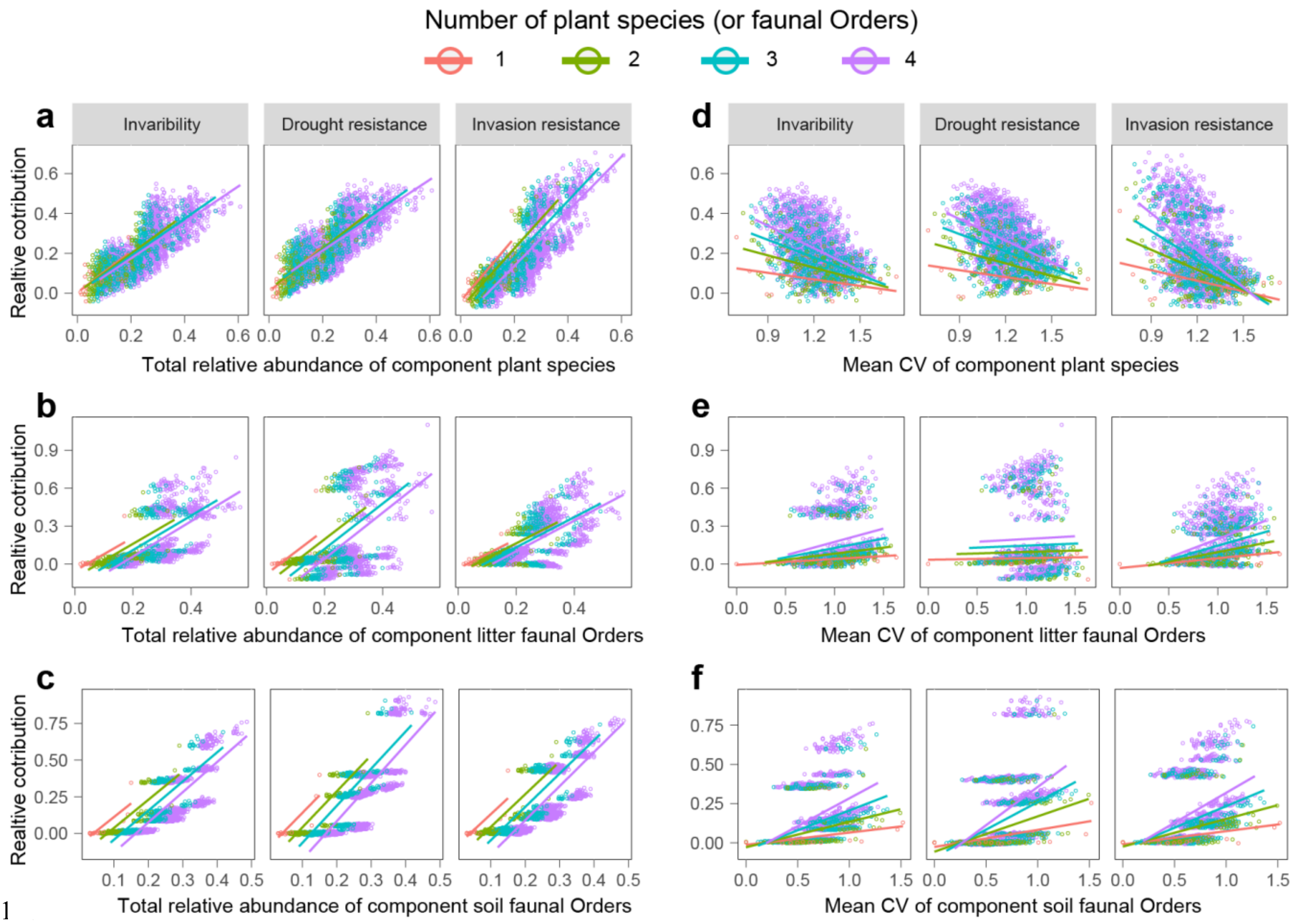

Supplementary Figure 5 Relative contribution of single (1) or a group of (2, 3, and 4) plant species (or faunal Orders) to the relationship between the stability of (**a, d**) plant, (**b, e**) litter fauna and (**c, f**) soil fauna community and the stability of ecosystem multifunctionality. X-axis: (**a-c**) total relative biomass (abundance) of single (1) or a group of component plant species (fauna Orders); (**d-f**) mean CV (coefficient of variance) of component plant species or faunal Orders. Y-axis: the relative contributions of plant species or faunal taxa were calculated as the percentage decrease of community-multifunctional stability relationship after the remove of plant species or faunal Orders, compared to the original relationship.
