# Supplementary material for: Faunal communities mediate the effects of plant richness, drought, and invasion on ecosystem multifunctional stability
Source: Commun Biol. 2022 Jun 1;5:527. doi: 10.1038/s42003-022-03471-0 (PMC9159989; doi:10.1038/s42003-022-03471-0)
Supplement: Supplementary file 5 — Description of Additional Supplementary Files [file 42003_2022_3471_MOESM5_ESM.pdf]

# Description of Additional Supplementary Files

**File name:** Supplementary Data 1
**Description:** Plant and faunal taxonomies, representative markers and accessions.
